# The Inhibitory Effects of Bioactive Compounds of Tomato Juice Binding to Hepatic HMGCR: *In Vivo* Study and Molecular Modelling

**DOI:** 10.1371/journal.pone.0083968

**Published:** 2014-01-02

**Authors:** Inmaculada Navarro-González, Horacio Pérez-Sánchez, Gala Martín-Pozuelo, Javier García-Alonso, Maria Jesús Periago

**Affiliations:** 1 Dept. of Food Science and Nutrition, Faculty of Veterinary Science. University of Murcia, Campus de Espinardo. Regional Campus of International Excellence "Campus Mare Nostrum", Murcia, Spain; 2 Computer Science Department, Catholic University of Murcia (UCAM), Murcia, Spain; National Cancer Institute, United States of America

## Abstract

The hypocholesterolemic effect of tomato juice has been investigated in an intervention study with rats, along with the possible inhibition effect of bioactive tomato compounds binding to the HMGCR enzyme. Two experimental groups (n = 8 Sprague-Dawley rats) were fed *ad libitum* for five weeks, with water or tomato juice provided to the control and intervention groups, respectively. Total, LDL and HDL cholesterol, and total triglycerides were analysed in plasma, and the lycopene content and the expression and activity of the enzyme HMGCR were determined in liver samples. A computational molecular modelling was carried out to determine the interactions between HMGCR and lycopene, chlorogenic acid and naringenin. Total, LDL and HDL cholesterol were significantly lower in the intervention group after the intake of tomato juice. In addition, a significant reduction in HMGCR activity was observed, although this was not accompanied by changes in gene expression. The molecular modelling showed that components of tomato can bind to the active site of the enzyme and compete with the ligand HMGCoA. Lycopene, from tomato juice, accumulates in the liver and can inhibit the activity of the rate-limiting enzyme of cholesterol biosynthesis, HMGCR.

## Introduction

Tomato fruits contain a related set of acyclic C_40_ carotenoids, predominant among which is lycopene, which is responsible for the deep red color of fully ripe tomatoes. The central carbon chain of alternating single double bonds in the lycopene series is 11 conjugated bonds in length, and confers the greatest singlet oxygen quenching ability of all tested carotenoids [1]. In addition, tomatoes contain other bioactive antioxidant compounds such as vitamin C and E, and phenolic compounds [2]. Of note, too, is the high content of hydrocinnamic acids (mainly caffeic acid and its ester chlorogenic acid) and flavonoids such as rutin and narigenin [3], [4].

Several studies have indicated a relationship between the consumption of tomato and tomato products (tomato juice, ketchup, tomato sauce, paste, soup, etc) and the prevention of some chronic diseases, including cardiovascular diseases (CVD) [5], [6], [2], [7]. After a systematic review of the literature, Mordente et al. [8] reported that more than sixty epidemiological studies have investigated the association between lycopene and CVD. Several *in vivo* and *in vitro* studies in human, animals and cell-culture models have suggested that lycopene is the phytochemical responsible for the beneficial effect related to the prevention of cardiovascular diseases [8], [9], but other bioactive compounds, such as the main phenolic compounds (chlorogenic acid, caffeic acid, rutin and naringenin), folates and antioxidant vitamins (E and C) could act synergistically in modulating inflammation and as oxidative stress biomarkers [10].

The role of lycopene in the prevention of cardiovascular diseases has been widely reported, the literature suggesting a variety of mechanisms for the inhibitory effect on pathogenesis and the progression of CVD. The biochemical mechanisms underlying the preventive effect of lycopene appear to be multi-factorial and include: 1) lowering the content of LDL-cholesterol [11], [12], 2) acting as antioxidant, decreasing the LDL oxidation and lipid peroxidation [12], [13], [10], [14], 3) modulation of inflammatory responses, by reducing the cytokines involved in CVD [15], [10], [14], and 4) decreasing total cholesterol [6], [16], [10], [13].

Some authors have described that lycopene can inhibit 3-hydroxy-3-methylglutaryl-CoA reductase (HMGCR) activity (EC 1.1.1.34) [11] or, at least, regulate its expression [17]; hence, lycopene can be considered a hypocholesterolemic agent since it modulates the activity of HMGCR, an enzyme involved in the cholesterol biosynthetic pathway. Moreover, in cultured macrophages, the inhibition of HMGCR activity by lycopene was similar to the effect mediated by statins [11], [18] mention that lycopene may be considered as dietary alternative to decrease statin doses in patients with slightly elevated cholesterol levels, reducing the side effects of this drug. In addition to lycopene, phenolic compounds have also been considered as hypocholesterolemic agents [19]. The main phenolic compounds of tomatoes, chlorogenic acids, rutin and naringenin [4], are dietary antioxidants which provide beneficial effects against CVD by decreasing total cholesterol, lipid peroxidation and inflammation [19], while also reducing the activity of HMGCR [20], [21].

These studies suggest that supplementation of the diet with lycopene, chlorogenic acid and naringenin could provide a moderate hypocholesterolemic effect due to the inhibitory action on HMGCR. Although, several hypotheses have been proposed to explain its action mechanism, the way in which lycopene exercises its biological activities is still unknown. The aim of the present study was to investigate the hypocholesterolemic effect of tomato juice in an intervention study with rats and to elucidate the possible mechanisms of the main bioactive compounds of tomato juice (lycopene, chlorogenic acid and naringenin) in relation with the activity and gene expression of the HMGCR enzyme in rat liver. In addition, the interaction of these compounds with the active site of HMGCR enzyme was evaluated, by computational molecular modelling.

## Materials and Methods

### Tomato juice

Tomato juice, obtained by an industrial standard process and retailed in glass bottles, was provided by Juver Alimentación S.L. (Cabezo de Torres, Murcia; Spain).The total content of bioactive compounds, including lycopene, folates and phenolic compounds, was analysed in tomato juice following the methods described in previous research studies [3], [4].

### Animals

Sixteen male Sprague-Dawley rats (8 weeks old and weighing 225–250 g) were obtained from the Animal Research Centre of Murcia University. The rats were maintained under controlled conditions of temperature (22°C) and air humidity (55%) and a 12 h light-dark cycle for two weeks before starting the experiment. During this period they had free access to the standard laboratory diet (Teklad Global 14% Protein Rodent Maintenance diet, Harland Laboratories) and tap water. The animal study was carried out under appropriate guidelines and was approved by the Bioethics Committee of Murcia University.

### Experimental design

After a two-week adaptation period, the animals were randomly divided into two groups before being placed in metabolic cages with identical environmental conditions. The two experimental groups were fed the standard laboratory diet mentioned above, but with different fluids to drink. The control group was given tap water and the intervention group tomato juice. For a period of five weeks, the animals were given free access to food and drink. Food intake was measured daily and the rats were weighed at weekly intervals. The mean value of daily lycopene intake was estimated in the intervention group, and the apparent absorption of lycopene was calculated taking into consideration the mean value of lycopene ingested with the tomato juice and the amount of lycopene excreted in faeces. The amount of excreted faeces and the volume of excreted urine were recorded daily, and samples were kept at −80°C for further analysis. At the end of the experiment, all rats were deprived of food overnight, anesthetized with isofluorane, and sacrificed using an intraperitoneal injection of sodium pentobarbital. Blood samples and livers were collected from the animals. Liver samples were immediately cut into small pieces and frozen with liquid nitrogen, before being stored at −80°C until the analytical procedures were carried out.

### Analyses of plasma

Blood samples were transferred into heparin-containing tubes. Plasma was obtained by centrifugation (3000 *g*, 10 min, 4°C) and the concentrations of total cholesterol, HDL and LDL cholesterol, and total triglycerides were analysed using an automatic analyzer (AU 600 Olympus Life, Germany).

### Analysis of lycopene in faeces and liver

Lycopene was analysed by HPLC, following a method adapted from Böhm [22]. Samples were defrosted and 0.5 g was homogenized with 400 mg of MgO, 300 µL of internal standard (astaxanthin, 100 ng/µL) and 35 mL of methanol/tetrahydrofuran (1/1, v/v) containing 0.1% butylated hydroxytoluene using a TissueRuptor (Quiagen, Duesseldorf, Germany). The resulting solution was vacuum filtered through Whatman^™^ grade No 5. filter paper (Whatman, England). The extraction was repeated twice more until the residue was colorless and the combined extracts were dried under vacuum at 37°C in a rotatory evaporator (Heidolph Laborota 4002-control). The residue was resuspended in ethanol, until the solution reached the defined volume of 10 mL and reduced to dryness in a vacuum. Finally, the residue was resuspended in 2 mL of ethanol and centrifuged at 20.817 *g* for 10 min at room temperature. Samples were filtered and analyzed by HPLC, using 1.3 mL min^−1^ methanol (Solvent A) and methyl tert-butyl ether (MTBE; Solvent B) and a gradient method at 17°C (Thermostat with Peltier cell model MFE-01, Análisis Vínicos, Villarrobledo, Spain) on a C30-column (250×4.6 mm, 5 µm, Trentec, Gerlingen, Germany). The gradient elution started with 90% A and 10% B to reach 55% A at 35 min, 40% at 40 min, then isocratic for 10 min and finally reaching 90% A at 60 min. *all-(E)-*Lycopene and its (*Z*)-isomers were quantified at 472 nm. Since standards of lycopene *(Z)*-isomers were not available, they were tentatively identified based on the retention times and absorption spectrum characteristics described in the literature [23].

### Analysis of phenolic compounds in rat liver

**Figure 1:**
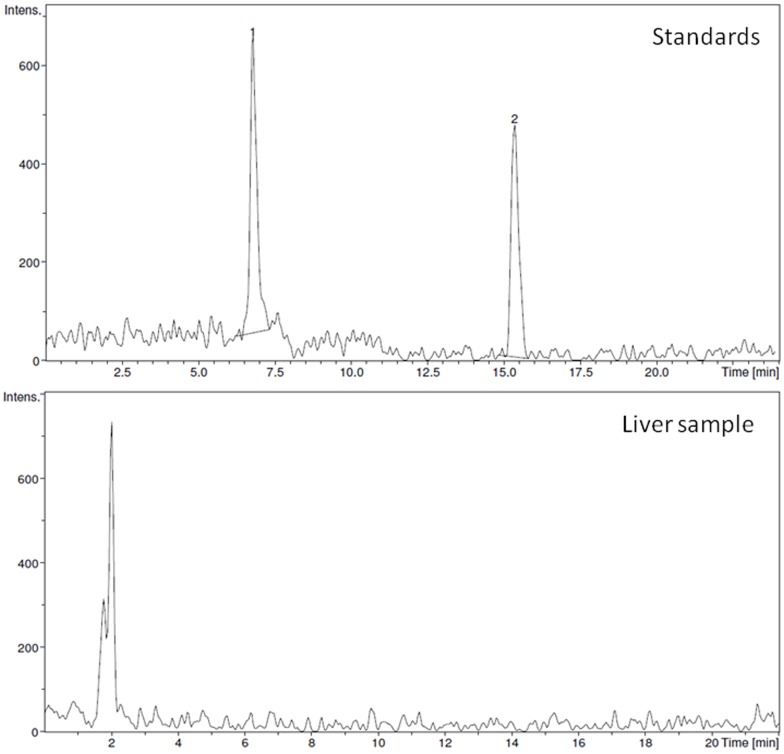
Chromatograms of standards and rat liver samples obtained in the HPLC analysis of chlorogenic acid (1) and naringenin (2). The images were similar in both control and intervention groups.

One hundred milligrams of freeze-dried livers were extracted with 1.5 mL of 70% methanol: water. The extraction was repeated two times and the combined extracts were dried in a vacuum concentrator (Eppendorf model 5301, Hamburg, Germany) until 1 mL. This volume was filtered through a filter of 0.22 µm and the sample was injected into HPLC equipment to detect and identify the main phenolic compounds of tomato juice in liver samples according to the method described by [24]. HPLC was carried out using a 150 µm×4.6 mm i.d. 5 µm C_18_ SunFire (Waters, Milford, PA, USA), maintained at 25°C and eluted at flow rate 1 mL/min with a 64 min gradient of 10–70% acetonitrile in 0.1% formic acid. The identification of chlorogenic acid and naringenin (Figure 1), was carried out with a Agilent 1100 HPLC system connected to an ION-Trap VL-01036 liquid chromatograph–ion trap mass detector equipped with an electrospray ionization (ESI) device (Agilent Technologies, Waldbronn, Germany). The mass spectrometer was operated in negative ionization mode. Analyzes were carried out using full scan, data dependent MS^2^ scanning from m/z 50 to 500. The capillary temperature was 350°C and the nebulizer was set at 60.00 psi. The auxiliary gas flow was set at 9.00 L/min. The identification was made using all the target ions resulting from the fragmentation of the ion m/z 353 of chlorogenic acid and ion m/z 271 of naringenin.

### Determination of HMGCR activity

The HMGCR enzyme activity of liver was determined by the colorimetric method described by Venugopala and Ramakishnan [25], which estimates the hydroxymethylglutaryl-CoA/mevalonate (HMGCoA/mevalonate) ratio as an index of the activity of HMGCR. HMGCoA was determined by reaction with hydroxylamine at pH 5.5 and subsequent colorimetric measurement of the resulting hydroxamic acid by formation of complexes with ferric salts. The mevalonate content was determined by reaction with the same reagent but at pH 2.1, a value at which the lactone form of mevalonate readily reacts with hydroxylamine to form hydroxamate. To prepare the liver homogenates, 0.5 g of rat liver was homogenized with 5 mL of saline arsenate solution (1 g of sodium arsenate per liter of physiological saline). Equal volumes of 10% tissue homogenate and diluted perchloric acid (50 mL/L v/v) were mixed, gently shaken for 5 min, and centrifuged (2000 rpm, 10 min). 2 mL of supernatant was treated separately in two test tubes with 0.5 mL of freshly prepared hydroxylamine reagent (alkaline hydroxylamine reagent to quantify the content of HMGCoA and hydroxylamine acid to quantify the mevalonate) and gently mixed. After 5 min, 1.5 mL of ferric chloride reagent was added, and after 10 min the absorbance was read at 540 nm, using a saline arsenate solution similarly treated as blank. The activity of HMGCR was estimated as the ratio between the absorbance readings of the HMGCoA and mevalonate test tubes.

### RNA extraction

Total RNA was extracted and isolated from a frozen liver using GeneElute Mammalian Total RNA Miniprep Kit (Sigma-Aldrich, St. Louis, MO, USA). The quantity and quality of RNA was assessed using a Picodrop Microliter UV/Vis Spectrophotometer (Picodrop Limited, Saffron Walden, United Kingdom). To eliminate possible trace amounts of genomic DNA in RNA preparations, the RNA was treated with DNaseI (Sigma-Aldrich, St. Louis, MO, USA). RNA samples were used in the qPCR (quantitative real time PCR) to assess the expression of HMGCR.

### Quantitative Real Time PCR (qPCR)

2 µg of total RNA from each tissue sample were reverse transcribed into cDNA (Applied Biosystems Foster City, CA) following the manufactureŕs instructions. Samples were used in the qPCR to assess the expression of HMGCR gene. The qPCR experiments were carried out using the 7500 Fast Real-Time PCR system (Applied Biosystem, Foster City, CA). The TaqMan primers and probes and TaqMan® Gene Expression Master mix (rat HMGCR) were from Applied Biosystem. The qPCR data for the tissues was normalized relative to the abundance of a validated endogenous control rat β-actin (4352931E) from Applied. Reactions were allowed to proceed in a total volume of 20 µL using 9 µL of cDNA (diluted 1∶10), 10 µL TaqMan Gene Expression Master Mix (2×) and 1 µL of TaqMan Gene Expression assay (20×) or TaqMan endogenous control (20×). The mean sample threshold cycle (*C*_T_) and mean endogenous control *C*_T_ for each sample were calculated from duplicate wells. The relative amount of target gene expression for each sample was assessed using a ΔΔC_T_ method [26]. In all experiments, appropriate negative controls containing no template cDNA were subjected to the sample procedure to exclude or detect any possible contamination, e.g. by genomic DNA.

### Molecular Modelling

In order to study interactions at molecular level between human HMGCR and the main bioactive compounds of tomato juice (lycopene, chlorogenic acid and naringenin), docking simulations were performed, since their predictions can reveal useful information [27] about the presence or absence of protein-ligand interactions and about how these interactions are established (electrostatic, van der Waals, hydrogen bonds, hydrophobic, etc), and concerning which residues of the protein are involved. In order to compare the interaction between these bioactive compounds and the enzyme HMGCR, and the binding capacity to the enzyme of the statins drugs, cerivastatin was included in the docking simulations.

The docking of lycopene, chlorogenic acid and naringenin to the prepared model of HMGCR and detailed binding energy calculations were performed with the Lead Finder v 1.1.10 software [28] using default configuration parameters. The reference ligand for mapping the inhibitor binding site was taken from the PDB structure 1DQ9. The size of the grid box for ligand docking was set to extend 30Å in each direction from the reference ligand. The dG-score produced by Lead Finder was taken as the predicted value of the ligand binding energy. Only the top-ranked poses were used for structural and energy analyses.

The full-atom model of HMGCR used in this study was prepared from the raw PDB structure, 1DQ9, by removing water molecules, adding hydrogen atoms, assigning the ionization states of the amino acids by using Protonate3D function of the MOE software package (Chemical Computing Group Inc., Montreal, Canada) and by calculating atomic partial charges using the AMBER99 forcefield [29] implemented in MOE. The HMGCR model was validated by docking of its cognate ligand (HMGCoA) and measuring the root mean square deviation (RMSD) of the docked ligand from its crystallographic position. The model revealed an RMSD <2Å. Next, redocking of the lycopene ligand from the PDB structure 1LGH was performed, giving an RMSD <2Å was obtained. These redocking data indicated the appropriateness of Lead Finder for use in the molecular docking studies in our context.

### Statistical analyses

For the analytical measurements of plasma, faeces and liver the analyses were conducted in triplicate and the data are expressed as mean ± SD of the results obtained for the 6 animals of each intervention group. A “Student-t” test was applied to determine significant differences in all the analysed parameters. The statistical analysis was conducted by SPSS Program ver. 18.0.

## Results

Table 1 shows the bioactive compounds contents of tomato juice. As can be seen, lycopene represents about 80–90% of total carotenoids in raw tomatoes and tomato products. The tomato juice showed a mean lycopene content of 108.1 mg/kg, with only 5% *(Z)-*isomers. The main individual phenolic compounds were chlorogenic acid (21.9 mg/kg), rutin (21.2 mg/kg), and naringenin (6.4 mg/kg).

**Table 1:** Lycopene, major phenolic compounds and folate contents of tomato juice^a^.

Bioactive compounds of tomato juice	
Total lycopene (mg/kg)	108.1±1.9
all*-(E)*-lycopene (mg/kg)	102.6±1.5
*(Z)*-lycopene (mg/kg)	5.4±0.3
Chlorogenic acid (mg/kg)	21.9±0.7
Rutin (mg/kg)	21.2±0.8
Naringenin (mg/kg)	6.4±0.3
Total Folates (mg/kg)	0.3±0.09

^a^ Data are expressed as mean ± SD of three determinations.

Table 2 shows food and drink intakes, the excreted faeces and urine, the lycopene intake, its apparent absorption and the concentration of lycopene, chlorogenic acid and naringenin in rat livers of the control and intervention groups. Diet consumption during the study period was recorded daily, but only the mean values are shown (g of feed per day). The animals in the two groups were fed *ad libitum* with the same diet and no significant differences in the amounts of ingested feed, or in the amount of water or tomato juice were observed, which explains why no differences were found in the amount of daily excreted faeces and urine. Tomato juice provided a mean of 3.5 mg of lycopene/day in the intervention group, and, according to the amount of lycopene excreted in faeces, the apparent absorption of this carotenoid was 44.5%. The lycopene was accumulated in the liver mainly as (all*-E)-*lycopene (2.5 µg/g), whereas the concentration of *(Z)*-isomers in the liver was 0.4 µg/g, representing 12% of total lycopene of the liver. Neither of the main phenolic compounds, chlorogenic acid and naringenin, was detected (Figure 1).

**Table 2:** Food and drink intake, excreted faeces and urine, lycopene intake, lycopene apparent absorption, lycopene concentration in liver and chlorogenic acid and naringenin content in liver (Control group: standard diet and water; Intervention group: standard diet and tomato juice)^a^.

Parameters	Control group	Intervention group
Food intake (g/day)	21.1±0.8	19.5±0.4
Drink intake (mL/day)	28.8±2.4	37.1±2.1
Excreted faeces (g/day)	8.60± 0.4	8.4±0.1
Excreted urine (mL/day)	10.1±1.4	11.9±0.3
Lycopene intake (mg/day)	−	3.5±0.1
Lycopene apparent absorption (%)	−	44.5±2.6
Liver all-(*E*)-lycopene (µg/g)	nd	2.5±0.60*
Liver (*Z*)-lycopene (µg/g)	nd	0.4±0.05*
Liver chlorogenic acid	nd	nd
Liver naringenin	nd	nd

^a^ Data are expresses as mean ± SD; nd: not detected

Significant differences p<0.05

Total cholesterol, LDL-cholesterol, HDL-cholesterol and total triglycerides were determined in plasma in both groups at the beginning and at the end of the intervention period (Table 3). The initial values for the plasmatic lipids were the same in both groups, since animals were maintained under the same conditions and with free access to feed and water. However, by the end of the intervention, total cholesterol and its fractions, LDL and HDL cholesterol, had decreased significantly (p≤0.05) in the intervention group, the differences to the control group levels being statistically significant. For total triglycerides, no significant differences were observed between groups and times.

**Table 3:** Initial and final levels of plasmatic lipids in both experimental groups (Control group: standard diet and water, Intervention group: standard diet and tomato juice)^a^.

Parameters	Control group	Intervention group	Significance
Initial Total Cholesterol (mg/dl)	105.0±3.3	105.0±3.3	1.00
Final Total Cholesterol (mg/dl)	99.2±4.1	80.9±6.5	<0.05*
Initial LDL Cholesterol (mg/dl)	22.0±1.4	22.0±1.4	1.00
Final LDL Cholesterol (mg/dl)	25.5±1.4	17.2±1.4	<0.05*
Initial HDL Cholesterol (mg/dl)	53.4±1.5	53.4±1.5	1.00
Final HDL Cholesterol (mg/dl)	54.4±2.3	44.3±3.5	<0.05*
Initial Triglycerides (mg/dl)	96.0±4.4	96.0±4.4	1.00
Final Triglycerides (mg/dl)	80.1±7.6	77.5±9.5	>0.05

^a^ All values are expressed as mean ± SEM (n = 8 per group).

Indicates significant differences (p<0.05) between groups.

Indicates significant differences (p<0.05) between initial and final values within each group.

**Figure 2:**
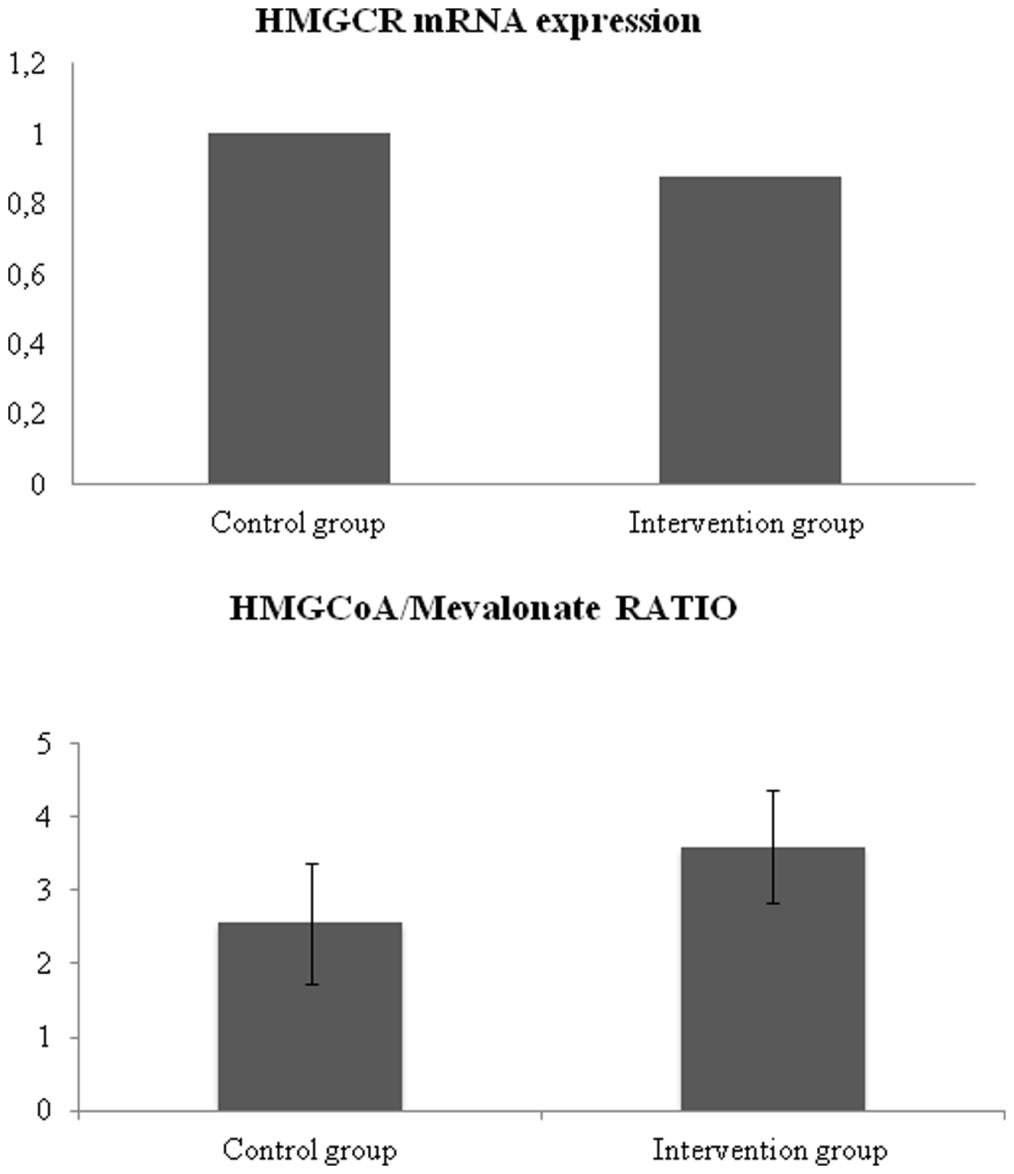
Relative HMGCR mRNA levels assessed by RT-PCR. The mRNA levels are expressed relative to β-actin gene (Figure 2a). HMGCoA/mevalonate ratio in liver tissue of male rats of both experimental groups (Figure 2b). (Control group: standard diet and water, Intervention group; standard diet and tomato juice). Data are expressed as mean±SD (* p<0.05 considered statistically significant).

Real time qPCR showed that the intake of tomato juice and the presence of lycopene in the rat liver did not significantly reduce the expression of HMGCR (Figure 2A). However, a significant decrease (p≤0.05) in the activity of this enzyme was observed in liver samples of the intervention group, as reflected by the increased HMGCoA/mevalonate ratio (Figure 2B).

**Figure 3:**
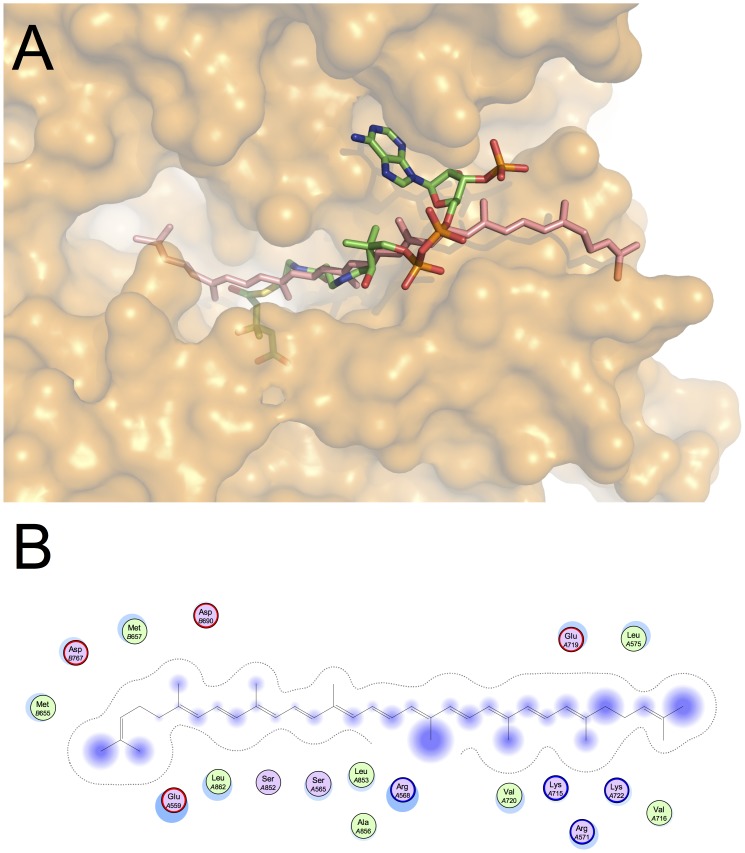
Docking results for lycopene (dark pink skeleton) in the active site of HMGCR (PDB ID: 1DQ9). (A) superposition with the crystallographic pose for HMGCoA (green skeleton), and (B) 2D diagram of protein-ligand interactions.

**Figure 4:**
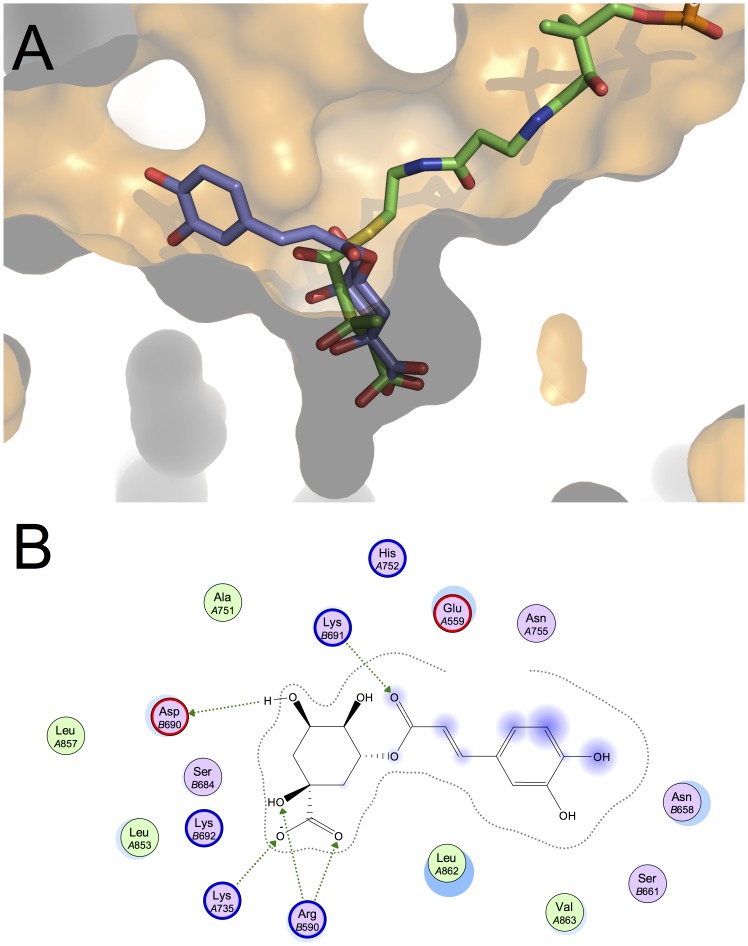
Docking results for chlorogenic acid (purple skeleton) in the active site of HMGCR (PDB ID: 1DQ9). (A) superposition with the crystallographic pose for HMGCoA (green skeleton), and (B) 2D diagram of protein-ligand interactions.

**Figure 5:**
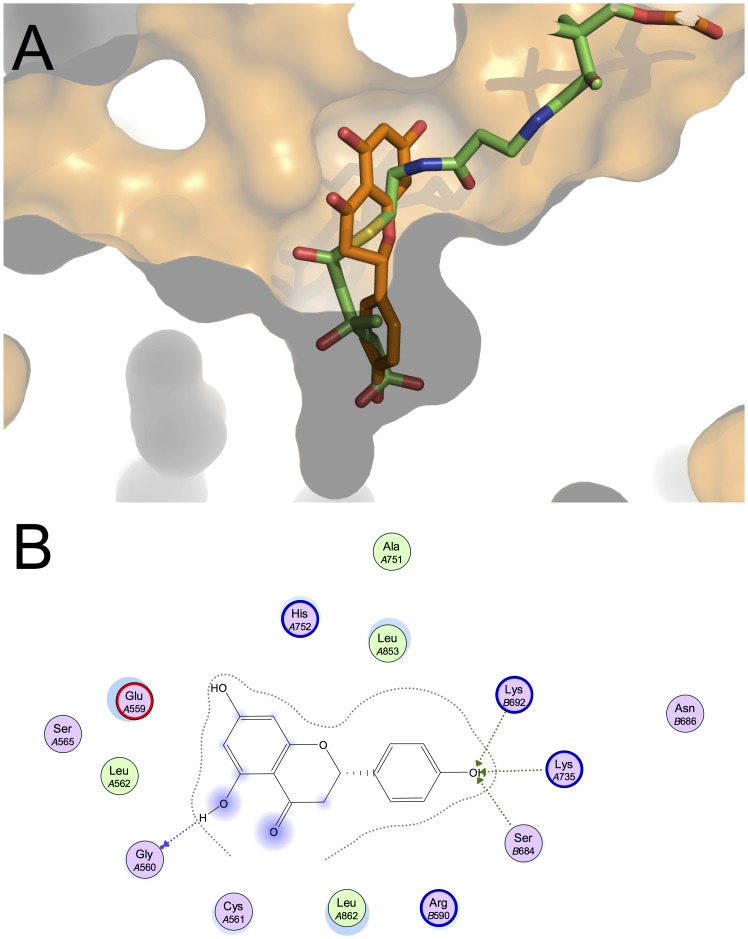
Docking results for naringenin (orange skeleton) in the active site of HMGCR (PDB ID: 1DQ9). (A) superposition with the crystallographic pose for HMGCoA (green skeleton), and (B) 2D diagram of protein-ligand interactions.

**Figure 6:**
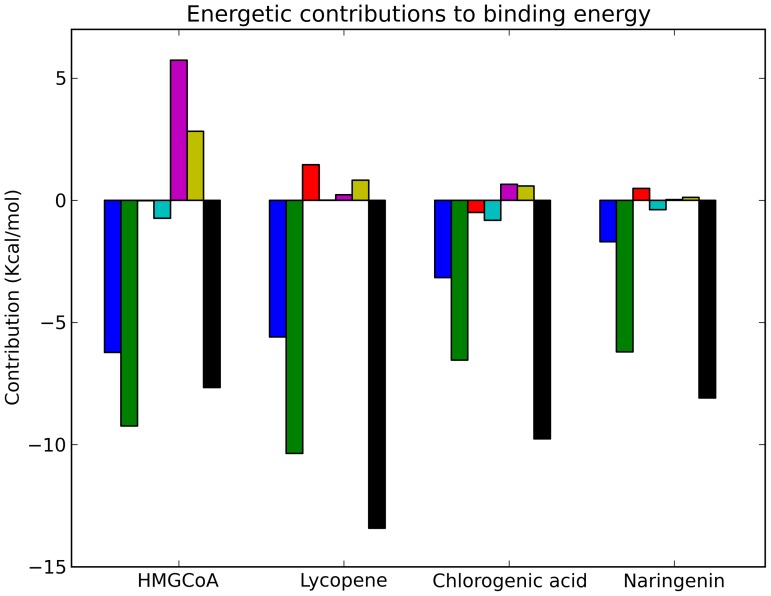
Energetic contributions (kcal/mol) to binding energy for ligands HMGCoA, Cerivastatin, Lycopene, Chlorogenic acid and Naringenin in their docking with HMGCR. Contributions (for each ligand, and from left to right) depicted are; van der Waals interactions (blue), solvation energy (green), hydrogen bonds (red), electrostatic energy (cyan), internal energy (magenta), energy of entropic losses associated with ligand's rotatable bonds (yellow) and total predicted binding energy (black).

The results of molecular modelling studies on the active site of the enzyme HMGCR and lycopene, cerivastatin, chlorogenic acid and naringenin are reported in Figures 3, 4, 5 and 6, and the total predicted energy binding of these interactions is summarized in Table 4. Molecular docking calculations with lycopene showed that it fitted tightly into the active site of HMGCR, where it was seen to bind with higher affinity than HMGCoA (Figure 3). This interaction suggests that lycopene competes with HMGCoA and inhibits mevalonate formation. Most of the stabilizing interactions for lycopene were seen to be van der Waals and hydrophobic in nature. The values obtained for the different energetic contributions to the binding energy of HMGCoA and lycopene are depicted in Figure 6. As can be seen, both HMGCoA and lycopene showed a similar degree of stabilization due to these van der Waals and hydrophobic interactions (blue and green bars), whereas HMGCoA had an internal energy penalty (magenta bar) given that, for HMGCoA to fit into the binding pocket, it must adopt a conformation that is far from its minimal energy conformation. However, lycopene fits easily into the binding pocket in a conformation very close to its minimal energy conformation. This internal energy difference might explain the higher affinity between the active site of HMGCR for lycopene compared with HMGCoA. Moreover, previous molecular docking studies reported a similar effect for lycopene in cyclooxygenases [30].

**Table 4:** Predicted and experimental ΔG values (Kcal/mol) obtained from molecular docking studies in HMGCR.

Molecule	Predicted ΔG	Experimental ΔG
HMGCoA	−7.7	−6.7*
Cerivastatin	−10.4	−11.4#
Lycopene	−13.4	—
Chlorogenic acid	−9.8	—
Naringenin	−8.1	—

Value obtained from Sarver et al., 2008.

^#^ Value obtained from Carbonell et al., 2005.

Chlorogenic acid and naringenin also fit tightly into the binding pocket of HMGCR, and, given to the presence of a multiple OH-group in the phenyl ring, they could establish hydrogen bonds with the active site of the enzyme. So, used by a hydrogen bond network is formed with most of the residues used in the 3-Hydroxy-3-Methyl-Glutaric (HMG) part of HMGCoA, as shown in Figures 4 and 5. It might be argued that chlorogenic acid and naringenin substitute the HMG moiety of HMGCoA in a similar way to that reported by other authors [31]. For example, a similar effect has been also described for the flavonoid conjugates from bergamot juice (brutieridin and melitidin), which seemed to invade the HMG-binding site, both compounds being good candidates for the inhibition of HMGCR [32].

To compare our docking predictions with well-known results for statins, we performed docking calculations with cerivastatin, using the PDB structure 1HWJ. Lead Finder v 1.1.10 software [28] predicted the binding pose of cerivastatin with an RMSD value of less of than to 1.5 Angstroms, the predicted binding energy being in close agreement with experimental results (see Table 4). Therefore, these molecular modelling predictions suggest that the main bioactive compounds of tomato juice compete with the reaction between HMGCR and HMGCoA (a summary of the predictions is shown in Table 4), thus inhibiting mevalonate formation, and consequently, reducing cholesterol synthesis.

## Discussion

Modern diets and eating habits are associated with unfavorable effects on the risk factors for cardiovascular diseases, since they can lead to increased circulating levels of cholesterol and triglycerides. In recent years, there has been considerable interest in the use of natural food components such as functional foods to treat hypercholesterolemia. In general, plant foods are considered beneficial for the prevention of cardiovascular diseases due to their low fat content and the presence of bioactive compounds with different chemical compositions, which may sometime be considered cholesterol lowering agents through different mechanisms [19]. The intake of tomato juice for five weeks by rats of the intervention group led to a significant reduction in total, LDL and HDL cholesterol, demonstrating a hypocholesterolemic effect. This effect has also been observed in human studies [6], [16], [10], [13] and has been associated with lycopene intake; however, the mechanisms of the hypocholesterolemic effect of lycopene have not been elucidated.

Tomato juice provides lycopene, chlorogenic acid and naringenin which can act synergistically in the reduction of plasmatic cholesterol levels. After absorption, lycopene is stored mainly in the liver. In this study, lycopene was accumulated in the liver in different isomeric forms (all*-E)* and *(Z)-*lycopene. The *(Z)*-isomerisation of lycopene *in vivo* takes place after absorption and inside the intestinal cells [33], so that the percentage of *(Z)*-isomers in the liver samples was higher than the percentage of *(Z)*-lycopene analysed in the tomato juice (12% *vs.* 5%). As regards chlorogenic acid, it is not well absorbed in the small intestine of rats [34], [35] but a small proportion can be quickly absorbed in the rat stomach in its intact form [36]. Due to its low bioavailability, the recovery of chlorogenic acid and its metabolites in urine did not account for more than 0.5% of the dose ingested by rats [34]. Recently, it has been seen that radioactive labelled [3-^14^C] caffeic acid is absorbed in the stomach where it is rapidly metabolised, while less than 0.1% of the total radioactivity intake was detected in the liver [24]. The bioavailability of naringenin in rats is also very low and depends on the amount of this flavonone in the diet, and on the glycoside moiety, which determines the delay of intestinal absorption [37]. In the present study, we did not analyse the metabolites of chlorogenic acid or naringenin in plasma and only investigated its presence in liver. Due to their low levels in tomato juice, compared with other foods and due to their low bioavailability and rapid metabolization, none of these compounds was detected in the rat livers of the intervention group.

Taking into consideration that the liver is involved in lipid metabolism, the presence of lycopene could be correlated with the cholesterol lowering effect observed in rats that drank tomato juice during the intervention period. The biosynthesis of cholesterol is catalyzed by HMGCR, which promotes the deacylation of HMGCoA to mevalonate [38]. The activity of HMGCR in animal cells has been shown to be sensitive to negative regulation by sterols and non-sterol products of the mevalonate pathway [39], [40]. In addition to sterols, lycopene can also reduce cholesterol synthesis in cell cultures by inhibiting HMGCR enzyme [11] or by decreasing its expression [17]. In agreement with these authors, we hypothesized two possible *in vivo* mechanisms to explain the effect of tomato juice intake on cholesterolemia: (I) a decrease in HMGCR activity, and (II) a reduction of HMGCR synthesis in the liver. In the current study the hypocholesterolemic effect was accompanied by a decrease in the activity of this enzyme in the rat liver, but not in its gene expression. The inhibition of HMGCR may involve a different mechanism; inhibition of the enzyme at a post-transcriptional level and competitive inhibitors of this enzyme. As the expression of HMGCR did not decrease in the rat liver, we postulate that lycopene may be a competitive inhibitor of this enzyme, since lycopene and other carotenoids are polyisoprenoids synthesized in plants from mevalonate via the HMGCR pathway [41]. This putative inhibitory mechanism was confirmed using the molecular modelling method, which showed that the HMGCR substrate-binding pocket accommodates lycopene molecules following competitive inhibition of the enzyme. Lycopene may interact with the active site HMGCR to form a complex similar to that formed between the substrate HMGCoA and the cerivastatin with the enzyme. Taking into account this finding, we propose that lycopene competes with HMGCoA, thus impeding mevalonate formation and consequently reducing cholesterol synthesis. The new information we provide concerning the mechanism of lycopene as a cholesterol lowering agent lends weight to the observations made in the scientific literature by several authors [11], [42], [18], based only on *in vitro* and *in vivo* studies. So, the daily intake of lycopene and it accumulation in the liver could exert a similar effect to that of statins, lowering total cholesterol by inhibiting HMGCR activity.

In addition to lycopene, chlorogenic acid and naringenin have also been considered cholesterol-lowering agents through the inhibition HMGCR enzyme. For example, Lee et al. [20] showed that naringenin lowers plasma and hepatic cholesterol concentrations by suppressing HMGCR and ACAT in rats fed a high-cholesterol diet supplemented with naringenin (0.1% w/w). In addition, chlorogenic acid, administered at 5 mg/kg body weight for 45 days to streptozotocin-nicotinamide induced type 2 diabetic rats, strongly reduced the activity of the above mentioned enzymes involved in lipid metabolism [21]. These *in vivo* studies reported an inhibition of the activity of HMGCR after the intake of phenolic compounds, but did not explain possible action mechanisms. The molecular modelling described in the present paper explains the formation of the complexes between the chlorogenic acid and naringenin, which would compete with the HMG moiety of the HMGCoA and show a similar effect to that of the flavonoids of bergamot juice [32]. Although these compounds would exert a hypocholesterolemic effect *in vivo* by inhibiting the activity of HMGCR, in our study the intake of chlorogenic acid and naringenin from tomato juice was very low (1.3 and 0.5 mg/kg of body weight, respectively).

Rai et al. [43] evaluated the effect of diallyldisulfide analogs from garlic on HMGCR using the same methodologies, and observed that these compounds are apparently more effective in reducing HMGCR mRNA than HMGCR activity. The mechanisms that can be ruled out in this respect include alteration of the enzyme and changes in catalytic activity mediated by phosphorylation [44]. Since lycopene and dyallydisulfide are chemically different, our results point to a different mechanism for the regulation of HMGCR by lycopene.

Furthermore, we postulate that the cholesterol-lowering effect is due to the inhibition of HMGCR activity associated with the accumulation of lycopene in the liver, the cholesterol metabolism is more complex, and cholesterol homeostasis is maintained through the coordinated regulation of pathways mediating cholesterol uptake, storage, de novo synthesis and efflux. The committed step in the biosynthesis of cholesterol and isoprenoids is catalyzed by the HMGCR enzyme, which is sensitive to negative regulation by both sterols and non sterols products of mevalonate pathways. This pathway produces numerous bioactive signaling molecules, including farnesyl pyrophosphate and geranylgeranyl pyrophosphate (GGPP), which can interact with different cellular receptors involved in the cholesterol metabolism [45]. *In vitro* studies with human macrophages have demonstrated that lycopene increases LDL receptor activity [11], and decreases the expression of HMGCR, reducing the activity of the enzyme and the activation of RhoA proteins, and increasing the expression of PPARγ, LXR and ABCA1 and Cav-1 [17] mediated by the concentration of the non-sterol mevalonate intermediate GGPP [45]. LXRs positively regulate several of the hepatic and intestinal genes required for cholesterol excretion from the body, including CYP7A, the rate-limiting enzyme for bile acid biosynthesis, and ATP binding cassette (ABC) genes involved in cholesterol transport in liver and intestine (ABCG5, ABCG8). In addition, LXRs directly and indirectly regulate the genes involved in fatty acid metabolism, including sterol response element binding protein-1c (SREBP-1c), fatty acid synthase, stearoyl-CoA desaturase, and acyl-CoA carboxylase, and also regulate the genes that control the secretion and metabolism of triglyceride-rich lipoproteins, including LPL, cholesteryl ester transfer protein, phospholipid transfer protein, and the apolipoprotein E/C-I/C-IV/C-II gene cluster [46]. Although we have not investigated the effect of lycopene on the expression of other key genes involved in the cholesterol metabolism, according to the above findings, inhibition of activity of the HMGCR in rat livers could regulate other proteins involved in the cholesterol metabolism, through a cascade mechanism mediated by LXR.

In conclusion, the cholesterol-lowering effect of tomato is mainly due to the lycopene content, since it is accumulated in the liver and can inhibit the activity of the rate-limiting enzyme of cholesterol biosynthesis, HMGCR. In this study we propose a novel putative molecular mechanism, showing that lycopene has a similar mechanism to statins. Hence, lycopene could enhance the effect of statins, reducing the dose of this drug in the treatment of hypercholesterolemia. Similarly, we have shown by molecular modeling that chlorogenic acid and naringenin can also be considered good candidates for use as HMGCR inhibitor agents; however, this effect is limited by their low bioavailability and by the fact that they are quickly metabolized. Moreover, due to the importance of mevalonate pathways in the regulation of cholesterol metabolism, further studies should be carried out to elucidate the *in vivo* mechanisms of lycopene that regulate plasmatic cholesterol through a cascade mechanism mediated by the inactivation of the HMGCR enzyme.
